# N-Acetylcysteine in Agriculture, a Novel Use for an Old Molecule: Focus on Controlling the Plant–Pathogen *Xylella fastidiosa*

**DOI:** 10.1371/journal.pone.0072937

**Published:** 2013-08-23

**Authors:** Lígia S. Muranaka, Thais E. Giorgiano, Marco A. Takita, Moacir R. Forim, Luis F. C. Silva, Helvécio D. Coletta-Filho, Marcos A. Machado, Alessandra A. de Souza

**Affiliations:** 1 Centro de Citricultura Sylvio Moreira, Instituto Agronômico, Cordeirópolis, São Paulo, Brazil; 2 Departamento de Genética e Biologia Molecular, Universidade Estadual de Campinas, Campinas, São Paulo, Brazil; 3 Departamento de Química, Universidade Federal de São Carlos, São Carlos, São Paulo, Brazil; University of the West of England, United Kingdom

## Abstract

*Xylella fastidiosa* is a plant pathogen bacterium that causes diseases in many different crops. In citrus, it causes Citrus Variegated Chlorosis (CVC). The mechanism of pathogenicity of this bacterium is associated with its capacity to colonize and form a biofilm in the xylem vessels of host plants, and there is not yet any method to directly reduce populations of this pathogen in the field. In this study, we investigated the inhibitory effect of N-Acetylcysteine (NAC), a cysteine analogue used mainly to treat human diseases, on *X. fastidiosa* in different experimental conditions. Concentrations of NAC over 1 mg/mL reduced bacterial adhesion to glass surfaces, biofilm formation and the amount of exopolysaccharides (EPS). The minimal inhibitory concentration of NAC was 6 mg/mL. NAC was supplied to *X. fastidiosa*-infected plants in hydroponics, fertigation, and adsorbed to organic fertilizer (NAC-Fertilizer). HPLC analysis indicated that plants absorbed NAC at concentrations of 0.48 and 2.4 mg/mL but not at 6 mg/mL. Sweet orange plants with CVC symptoms treated with NAC (0.48 and 2.4 mg/mL) in hydroponics showed clear symptom remission and reduction in bacterial population, as analyzed by quantitative PCR and bacterial isolation. Experiments using fertigation and NAC-Fertilizer were done to simulate a condition closer to that normally is used in the field. For both, significant symptom remission and a reduced bacterial growth rate were observed. Using NAC-Fertilizer the lag for resurgence of symptoms on leaves after interruption of the treatment increased to around eight months. This is the first report of the anti-bacterial effect of NAC against a phytopathogenic bacterium. The results obtained in this work together with the characteristics of this molecule indicate that the use of NAC in agriculture might be a new and sustainable strategy for controlling plant pathogenic bacteria.

## Introduction

*Xylella fastidiosa* is a Gram-negative bacterium that causes serious diseases in many economically important crops including citrus, grapevines, plums, almonds, peaches, and coffee [1]. In citrus plants, it is responsible for Citrus Variegated Chlorosis (CVC), a disease that has caused millions of dollars in damage to the Brazilian citrus industry [2]. Due to the worldwide importance of this phytopathogen, it was recently included as one of the ten most important plant pathogenic bacteria [3]. *X. fastidiosa* is restricted to the xylem of infected plants, and it is transmitted by insect vectors (sharpshooters) that feed from the xylem vessels. Once in the xylem, the bacteria cause disease as a probable result of vessel colonization through biofilm formation that blocks the flux of water and nutrients from the roots to the canopy [4–6]. In addition, cells in the biofilm are able to express many genes associated with pathogenicity, and adaptations in the plant may contribute to symptom development [7,8].

CVC symptoms start as yellow spots on the adaxial surface and develop to necrosis as the disease progresses. In addition, foliar wilt and zinc deficiency-like interveinal yellowing are observed. Severely affected fruits are small and hard and are unsuitable for the juice industry as well as the fresh fruit market. Yield losses of severely affected sweet orange trees can be as high as 60-80% [2].

The main control measures of plants with CVC are focused on i) avoiding the bacterial transmission to healthy plants though the use of insecticides to control the vector population, ii) production of seedlings and nursery plants in greenhouses to avoid early insect transmission, iii) pruning of affected branches with a low level of symptoms, and iv) the eradication of severely infected plants [9]. These combined measures have increased the cost of production, and studies focusing on the elucidation of bacterial signaling and its interaction with the plant and insect hosts have therefore been conducted with the aim of developing new strategies to control the occurrence and spreading of *X. fastidiosa* [6,9–15]. Transgenic plants are also a control strategy that has been used against *X. fastidiosa* [16]. Although it is an interesting strategy, the transgenic strategy will take a long time to be adopted by the growers; therefore, other more immediate strategies should be explored to control this bacterium.

The most accepted mechanism of pathogenicity of *X. fastidiosa* involving biofilm formation shares similarities with bacteria that cause human diseases such as *Pseudomonas aeruginosa, Staphylococcus aureus*, *S.* epidermidis, and *Escherichia coli* in which biofilm formation is an essential step for host colonization and disease development [17]. One common strategy to control bacterial human diseases is the use of antibiotics; however, this strategy is not used for controlling plant pathogens due to the high cost for field application, low efficiency, and risk of environmental contamination. Another approach used to control bacterial biofilms in human diseases is the use of N-Acetylcysteine (NAC). NAC is an analogue of cysteine that disrupts disulfide bonds in mucus and is one of the smallest drug molecules used in medicine [18–24]. This molecule is able to decrease biofilm formation of a variety of Gram-negative and Gram-positive bacteria and reduces the production of extracellular polysaccharide matrix while promoting the disruption of mature biofilms [18,22–29].

The antibacterial properties, the ability to inhibit biofilm formation, the low cost, and the lack of any known environmental damage make NAC a good candidate to be tested against *X. fastidiosa*. The effect of this molecule on a phytopathogen has never been studied before. Thus, we investigated the effects of NAC on the (i) biofilm formation and cell viability, (ii) production of extracellular polysaccharides (EPS), and (iii) CVC symptoms and the *X. fastidiosa* population in sweet orange (*Citrus sinensis* L. Osbeck) infected plants under different NAC application conditions.

## Materials and Methods

The effect of NAC on *X. fastidiosa* was evaluated in two different sets of experiments. We first tested its effect on *X. fastidiosa* grown in artificial medium (*in vitro* experiments) and then on bacteria in plants (*in planta* experiments), for which we used three different methods to treat the plants with the molecule.

### In vitro experiments

#### *X. fastidiosa* strain and growth conditions

*X. fastidiosa* strain 9a5c, isolated from CVC symptomatic sweet orange trees kept in a greenhouse, was grown in periwinkle wilt broth medium (PW) [30] at 28°C in a rotary shaker at 130 rpm for 7 days. Subsequently, an aliquot of bacterial suspension was transferred to a defined medium, XDM2 [31], and kept at the same growth conditions. An inoculum at 10^8^ CFU/mL was obtained after one week of growth. For the experiments with NAC (purchased from Sigma-Aldrich, ≥99% purity grade), different amounts of this substance were added to 18 mL of XDM2 medium and 3 mL of the bacterial inoculum in Erlenmeyer flasks. The NAC final concentrations were 1.0, 2.0, and 6.0 mg/mL (established after preliminary experiments – data not shown). The medium pH, after NAC addition, was maintained at 6.8 with 1 M NaOH. Controls without NAC were also included. After 14 days under agitation, the bacterial biofilms attached to the glass surface at the medium-air interface were collected. The remaining medium in each flask was also collected and centrifuged at 8,000xg for 5 min. The pellets, as well as the collected biofilms, were washed three times with Milli-Q water to eliminate any residual culture medium. The total biomass analysis was performed with both the cells in biofilms and cells from the pellets (planktonic cell fraction).

#### Total biomass and cellular viability

The attached biofilm was quantified by staining the remaining population with 1% crystal violet [32] for 30 min at room temperature. Excess crystal violet was removed by washing the stained cells three times with Milli-Q water. Crystal violet bound to the cells was recovered in 1 mL of absolute ethanol and quantified by measuring absorbance at 570 nm. The same procedure was performed for the planktonic cells. To quantify the living cells, an aliquot of 100 µL of each fraction (biofilm and planktonic) was used in a serial dilution in phosphate buffered saline (PBS). Each point of the serial dilution was plated in Petri dishes containing PW supplemented with albumin bovine serum. After 30 days, the number of colony forming units (CFU) in each plate was counted. We performed three biological replicates and used three plates for each point of the serial dilution per replicate. Statistical analyses were performed using a *t* test (*P* ≤ 0.05).

#### Quantification of exopolysaccharides (EPS)

The total amount of EPS was quantified using the phenol-sulfuric acid method [33], with a standard curve developed with different concentrations of glucose. We calculated a linear regression (y = 52.145x - 3.2503) and used it to interpolate the EPS amounts in mg/mL for each sample. Statistical analyses were performed using the *t* test (*P* ≤ 0.05) to compare the averages of the treatments.

#### Microscopy of X. fastidiosa biofilms under NAC treatments

For the microscopy analysis, biofilms were obtained using glass coverslips as a surface placed on the bottom of 24-well Nunclon, Delta SI Multidishes (Nunc). One milliliter of XDM2 liquid medium, 100 µL of an *X. fastidiosa* inoculum (optical density, 0.1), and a specific concentration of NAC (1.0, 2.0, 4.0, or 6.0 mg/mL) was added to each well. The pH of the medium was adjusted to 6.8. The culture plates were incubated statically at 28°C and were analyzed after 15 days of growth. The XDM2 medium was gently removed from the wells with a pipette, and the biofilm adhering to the cover glasses was washed as already described in the literature [34]. The biofilm cells were stained with Syto 9 (Invitrogen) diluted 1:600 in autoclaved Milli-Q water. Images were obtained using an oil immersion lens (numerical aperture, 1.0) with an Olympus BX61 fluorescence microscope. For Syto 9, a filter that permits excitation at wavelengths between 460 and 490 nm and emission at wavelengths between 500 and 520 nm was used. For each concentration of NAC, three replicates were analyzed. The controls were performed using the same conditions previously described but without the addition of NAC.

### In planta experiments: NAC in hydroponic systems

#### Plant inoculation and hydroponics setup

Symptomatic plants infected with *X. fastidiosa* were obtained through tongue approach grafting [35]. A branch segment from CVC-diseased sweet orange “Pera” (donor plant) and the stem from a healthy seedling (recipient plant) grown in 20-cm-tall plastic tubes were vertically sliced (4-5 cm longer) in such a way that they tongued into each other. The graft area was secured with tape until the graft union healed (Figure S1). Next, the top of the recipient plant was cut off just like the donor plant but keeping the branch of the donor plant almost 15 cm longer. Using this method, all *X. fastidiosa*-infected plants came from the same symptomatic donor plant.

The plants were then transferred to a hydroponic system consisting of Leonard jars [36] made with 750 mL glass bottles and painted on the outside with a metallic color to avoid light penetration (Figure S2). Autoclaved vermiculite and sand (1:1) were added to the top of the jars, and 250 mL of nutritive solution [37] containing different concentrations of NAC (0.48, 2.4 or 6.0 mg/mL) was added to the bottom. The nutritive solutions were replaced weekly. We performed two biological experiments in different periods of the year (May and December) with four replicates for each treatment. Controls grown with nutritive solution deprived of NAC were also analyzed. After sampling in the second experiment, we interrupted the NAC treatment and maintained the plants under greenhouse conditions for evaluating possible symptom resurgence. A summary of the analysis realized in each biological experiment is shown in Table 1.

**Table 1:** Summary of the performed analyses in each *in vivo* experiment.

**Analyses / Biological experiments**		**1**	2*
Concentration of NAC (mg/mL)		0.48 and 2.4	0.48, 2.4 and 6.0
Leaf symptoms of CVC		Yes	Yes
Leaf symptoms after interruption of NAC treatments		No	Yes
Bacterial quantification by qPCR	Time 0	Yes	Yes
	After 1 month	Yes	No
	After 3 months	Yes	Yes
Bacterial isolation		No	Yes
NAC quantification by HPLC		Yes	Yes

*After sampling in the second experiment, we interrupted the NAC treatment and maintained the plants under greenhouse conditions for evaluating possible symptoms resurgence.

#### Visual evaluation of leaf symptoms and bacterial quantification by qPCR

Every two weeks after beginning of treatment, we evaluated the progress of symptoms on the leaves (yellow spots and necrosis) and stored the data as photographic images. To verify the bacterial colonization in the plants, we estimated the number of bacterial cells before and after NAC treatments using TaqMan® real-time quantitative PCR as previously described [38]. The initial population (before treatment) was assumed to be 100%, and the final population was estimated as a percentage of the initial population. The values were plotted using a logarithmic scale for better visualization of the data.

Before bacterial quantification by qPCR, a standard curve was established using a reference weight of 2.94 x 10^-6^ ng for one genome of *X. fastidiosa*. A curve was performed with known amounts of bacterial DNA mixed with genomic DNA of sweet orange to mimic the condition of DNA extraction from the biological samples used in the experiment (*X. fastidiosa*-infected leaves and petioles). The range used in the curve was 10^2^ to 10^8^ cells/mg of plant tissue. The standard curve obtained [*y* = -3.3483x (number of DNA copies) + 38.196] showed a linear range with a correlation coefficient of 0.9955.

To perform the analysis, we randomly collected leaves that were located above the grafting point after 30 and 90 days and after 90 days in the first and second biological experiments, respectively. The total DNA from the petioles of leaves from the plants submitted to the different treatments (Table 1) as well as the negative controls was extracted using an Invisorb Spin Plant Mini Kit, quantified in a NanoDrop ND-1000 spectrophotometer (NanoDrop Technologies), and the amounts in each sample were standardized by concentration. The samples were analyzed in triplicate by qPCR using the ABI 7500 detection system (Applied Biosystems) [38]. No template controls were also included to detect any spurious signal arising from the amplification of contaminant DNA.

#### Bacterial isolation

For bacterial isolation, the midribs and petioles from the leaves in the second biological experiment were cut in small pieces, sterilized in 1% sodium hypochlorite and 70% ethanol (2 min each), and washed three times in autoclaved distillated water. These plant materials were then macerated in 1 mL of PBS, and the cell suspensions were used in serial dilutions. The *X. fastidiosa* populations were estimated by counting the CFUs grown on PW containing BSA [30].

#### NAC quantification by HPLC

The samples for HPLC analysis were collected from nutritive solution at the bottom of the Leonard jars and stored at -80^°^C until used. The analysis was performed using an Agilent Series 1200 Modular Liquid Chromatography apparatus (Agilent Technologies), configured with a G1322A degasser, G1311A quaternary pump, G1329A autosampler, G1316A column oven and G1314B UV detector. The control of the HPLC system, the acquisition and processing of data were conducted through Agilent Technologies EZCrom SI software (G6702AA, s.n. 08021502300). The reversed-phase procedure in isocratic mode utilized a stainless steel Phenomenex^®^ Luna C18 column (250x4,6 mm i.d., 5 μm particle size, Torrance, CA, USA) coupled with a Phenomenex^®^ C18 (4x3 mm i.d., 5 μm particle size, Torrance, CA, USA) security guard cartridge. The mobile phase consisted of a mixture of acetonitrile and formic acid 0.1% (40: 60 v/v). The column temperature was maintained at 35 °C, and the flow rate was 1.2 mL/min with an injection volume of 20 µL. All experiments were performed at 330 nm. Sample derivatization, calibration curve plotting and method validation were performed as described in the supplemental material (Text S1). Derivatization was carried out to improve the calibration sensibility of the method (Limit of Detection).

#### Study of NAC degradation rate in the nutritive solution

To verify if NAC was also being degraded in the nutritive solution, an experiment was performed with the same experimental condition used for the hydroponics experiments. In parallel with those experiments, we maintained three Leonard jars with 600 mg of NAC in 250 mL of nutritive solution without a plant to follow the environmental degradation. The jars were left in the greenhouse, and samples for HPLC analysis were collected from the solution after 1, 7, and 14 days. Evaluation of 7 days was necessary for experimental control and after that to verify if further NAC degradation occurred, with the aim of testing its availability time to the plant and possible behavior in field conditions.

### In planta experiments: NAC in fertigation system

#### Experimental conditions

Branches of sweet orange of the “Pera” variety with CVC symptoms were used for grafting into “Rangpur” lime rootstocks as described before. The plants were transplanted to 15 L vessels filled with commercial Plantmax substrate (Eucatex). These plants were maintained in a greenhouse for two years, and all of the new branches of the plants displayed CVC symptoms. The experiment was conducted with the following treatments: 5 plants without NAC (control), 6 plants with NAC added via fertigation and 6 plants with NAC added via fertigation plus injection in the trunk (Figure S3). For injection, the stems of the plants were drilled, and a syringe containing 10 mL of the NAC solution was inserted in the hole (Figure S3). The concentrated NAC solution was prepared using 45 g of NAC added to 1 L of water. The pH was adjusted to approximately 6.0-7.0 with 5 M NaOH. The solution was added to a fertigation tank containing 90 L of water, which was homogenized by mechanical stirring. The solution was replaced once a week. The fertigation system was programmed to release 0.5 L of this solution twice a day (each plant received 1 L of water, and then approximately 0.5 g of NAC was added per day). The control plants were irrigated by a similar system and also received 1 L of water (without NAC) per day.

#### Symptoms analysis and bacterial quantification of the infected plants before and during NAC treatment

The analysis of symptoms was performed by counting the chlorotic spots (characteristic of CVC symptoms) on every leaf of each plant before NAC treatment and every 3 months during the treatment (6 months in total). In addition to counting, some other features were also monitored, such as the size of the chlorotic spots and appearance of necrosis, to follow the disease development. For quantifying the bacterial population, 3 asymptomatic branches of each plant, surrounded by symptomatic branches, were selected and used for qPCR evaluations. Four young leaves were collected from the branches of each plant, totaling 12 leaves per plant. The leaves were collected along the whole extension of the branches, before and 6 months after beginning the NAC treatment. The bacterial population was estimated using the linear regression equation of the standard curve from the values obtained from the samples, as already described.

### In planta experiments: NAC adsorbed to organic fertilizer

#### Experimental conditions

Due to the promising results obtained in the fertigation experiment, a new experiment using CVC diseased plants was performed to test if NAC adsorbed to a fertilizer could be better absorbed by the plant root system. Three-year-old CVC symptomatic plants grown in 15 L vessels with substrate were pruned before the treatments with NAC to normalize the plant size as well as to eliminate all of the symptomatic leaves. NAC was adsorbed in the slow-release organic fertilizer Super Adubo® 2-10-10 (NAC-Fertilizer) in a process performed by the manufacturer of the fertilizer (www.agrolatino.com.br). The final amount of NAC was 2 g/Kg of fertilizer, and 300 g of the NAC-Fertilizer was added to each plant (Figure S4) at the beginning of the experiment, 4 and 7 months later. In addition, some plants were also sprayed with a NAC solution containing a commercial adjuvant (120 mg of NAC, 0.05 mL of Silwet L-77, in 50 mL of water, per plant) concomitant with NAC-Fertilizer application (Figure S4). This step was performed to check if there was an increased response when NAC was also applied to the leaves. The experiment was conducted in a greenhouse with the following treatments: 5 plants with CVC treated with NAC-Fertilizer; 5 plants with CVC treated with NAC-Fertilizer plus NAC sprayed on leaves, and 5 plants with CVC treated with the same fertilizer but without NAC. Additional controls such as two healthy plants treated with NAC-Fertilizer, 2 healthy plants treated with NAC-Fertilizer and spray, and 2 healthy plants treated with fertilizer without NAC were included to verify any possible effect of NAC on the plants.

#### Symptoms analysis and bacterial quantification of the infected plants before and during NAC treatment

Symptoms were monitored weekly in the plants by counting the number of symptomatic leaves and quantifying disease severity using a diagrammatic scale [39] (Figure S5). *X. fastidiosa* quantification was conducted via qPCR using the same methodology described previously. Both the disease progress and bacteria count were evaluated in 4 sections of randomly selected plant canopy. The same setup was used for all sampling performed throughout the experiment.

## Results

### In vitro experiments

#### NAC reduces biofilm formation, EPS amount and acts as an antimicrobial compound

**Figure 1:**
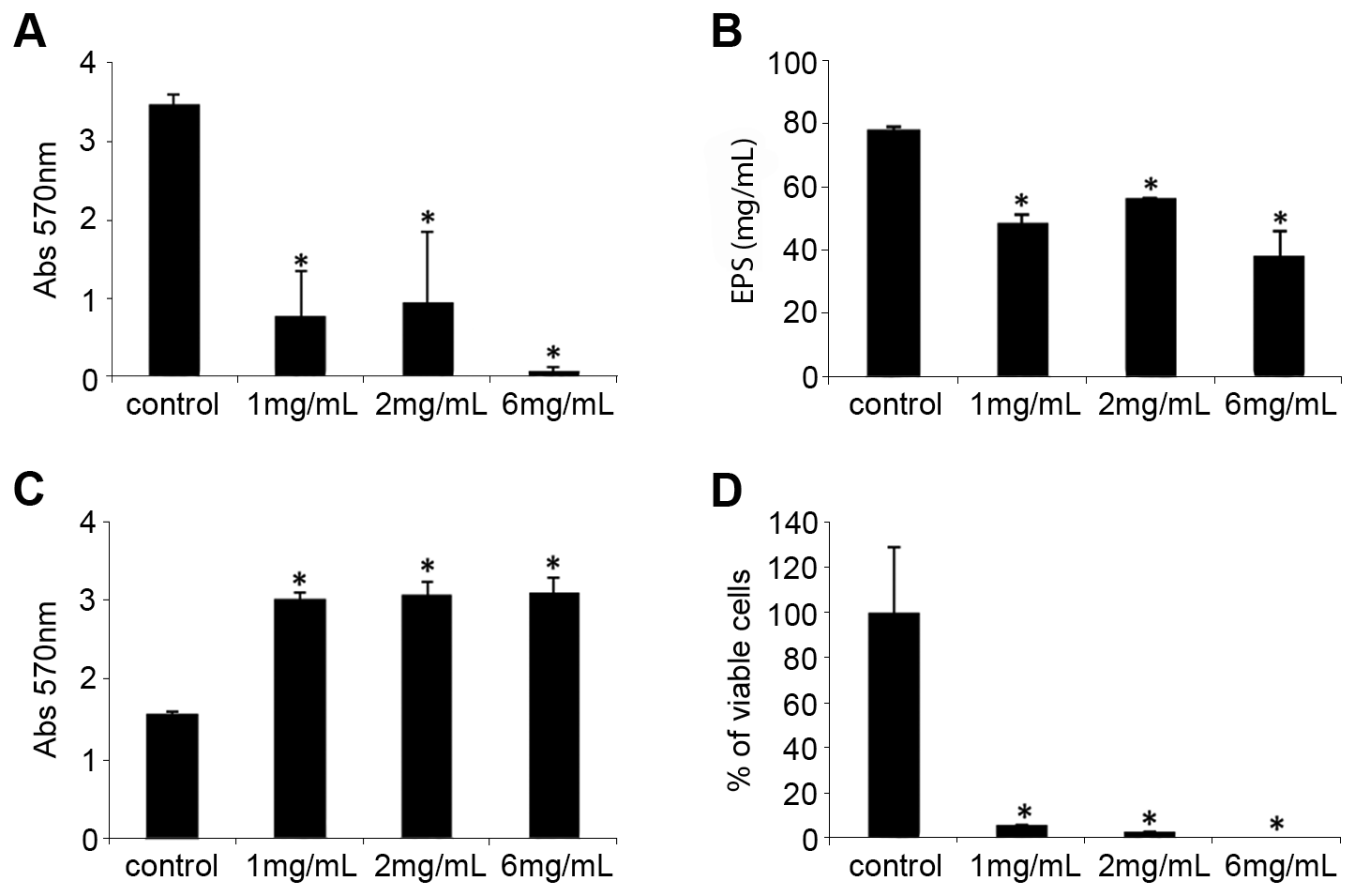
Effect of NAC on *X. fastidiosa* cells. NAC concentrations of 1.0, 2.0, and 6.0 mg/mL were used for treating the cells. The treated plants were compared with the untreated control plants. The treatments were compared in relation to biofilm formation (**A**), amount of EPS (**B**), total biomass of planktonic fraction (**C**), and cellular viability (**D**). * indicates significant difference compared with the control (*P* < 0.05). The values correspond to the mean of three biological replicates. Error bars indicate the standard errors of the means.

Concentrations of NAC higher than 1.0 mg/mL significantly decreased *X. fastidiosa* biofilm formation and the amount of EPS (Figure 1A-B). The biofilm reduction was a consequence of the inability of cells to adhere and form biofilm, resulting in a significant increase in the biomass of the planktonic fraction (Figure 1C). However, the majority of the cells in the biomass were dead, and this response was dose-dependent with no viable cells being observed with 6 mg/mL of NAC (Figure 1D). Thus, the increase of biomass in the planktonic fraction in the NAC treatment was due to an accumulation of dead cells, indicating that NAC also acts as an antimicrobial agent.

**Figure 2:**
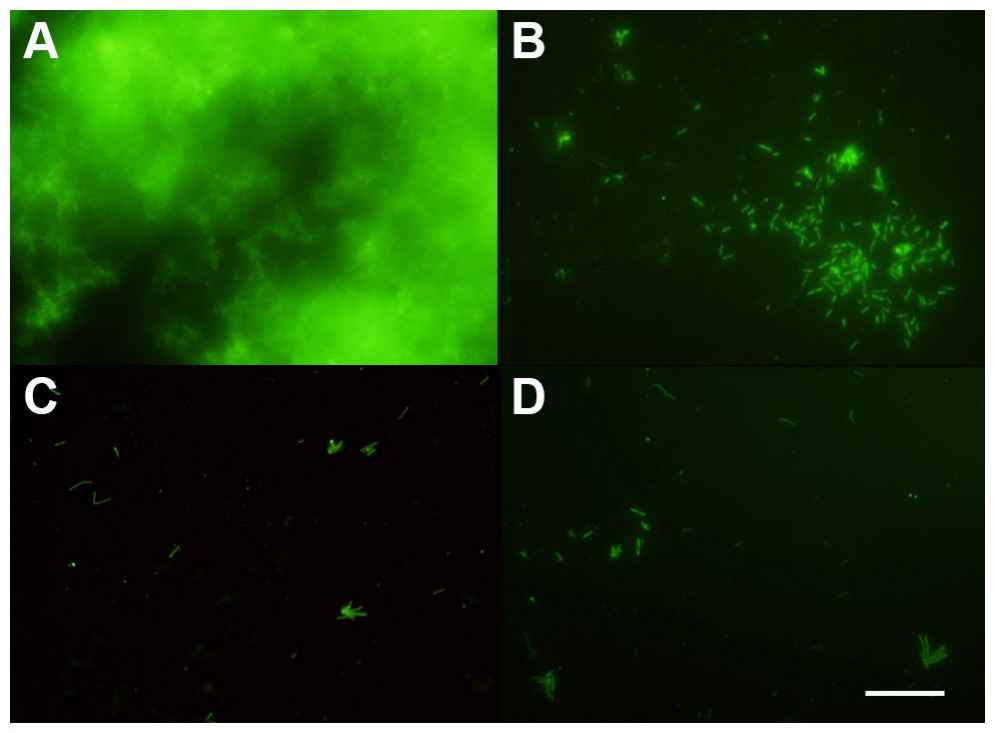
Fluorescence labeling of *X. fastidiosa* biofilm. Cells in biofilm were either not treated with NAC (**A**) or exposed to different NAC concentrations, 1.0 mg/mL (**B**), 2.0 mg/mL (**C**), and 6.0 mg/mL (**D**) and stained with Syto 9 to evaluate the characteristics of the formed biofilm (Scale bar, 20 µm).

Microscopy analysis was used to monitor the effect of NAC on *X. fastidiosa* biofilm development on cover glasses. As observed in Figure 2, the adherent *X. fastidiosa* community at 15 days after inoculation is characterized by a mature biofilm with a multilayer of cells at a high density (Figure 2A), but with 1.0 mg/mL of NAC, only small clusters of cells were visualized (Figure 2B), and at higher concentrations of NAC (2 and 6 mg/mL), only dispersed cells remained on the glass surface (Figure 2C-D). These results indicate that NAC negatively affected cell adhesion and, consequently, the biofilm formation in *X. fastidiosa*.

### In planta experiments

#### Plants treated with NAC present fewer Xylella fastidiosa cells in hydroponic systems

**Figure 3:**
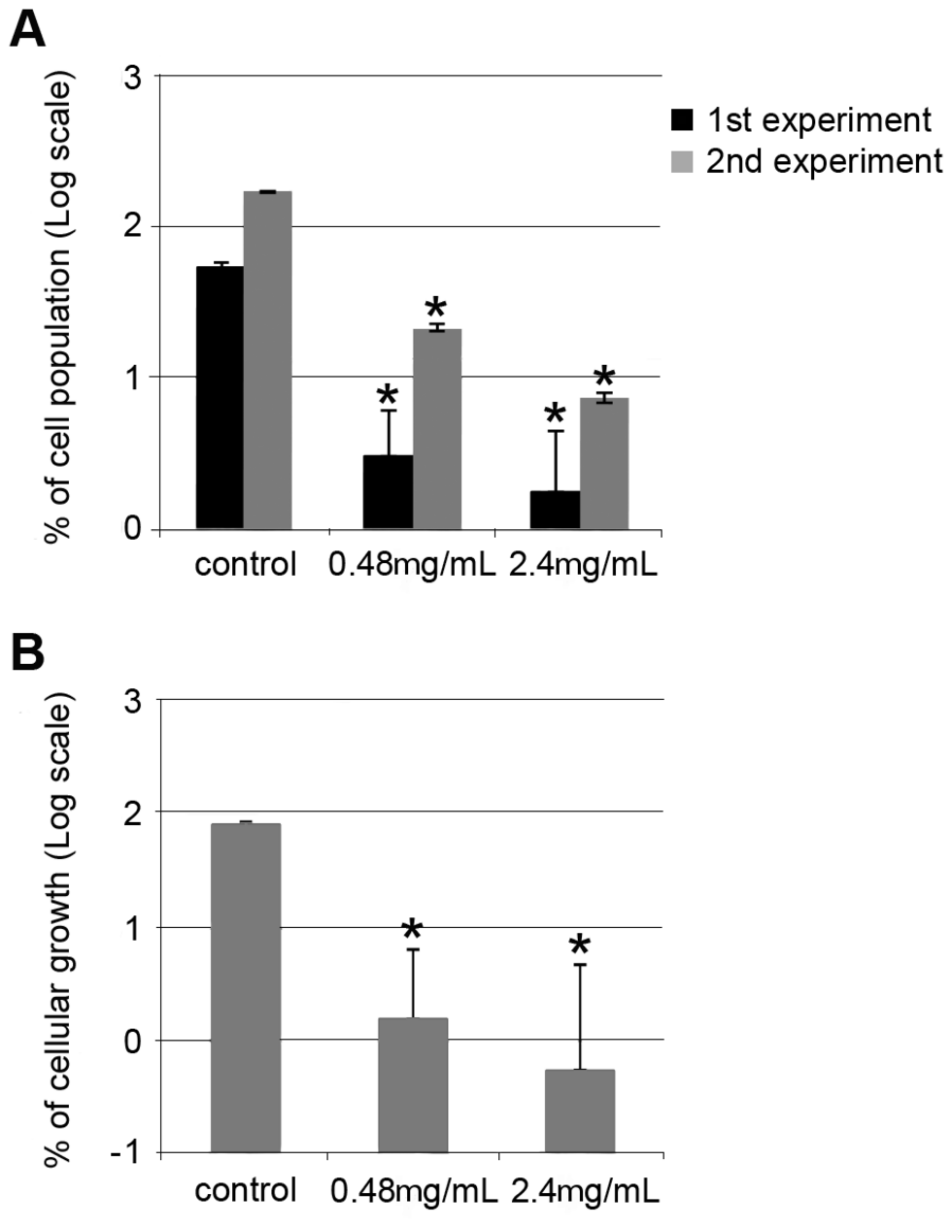
Effect of NAC on *X. fastidiosa* population in plants in hydroponics systems. Plants were treated with different concentrations of NAC, and the populations were monitored by real-time PCR analysis (A) and isolation in culture medium (B). The results are represented in log scale and refer to the percentage of the final population in relation to the beginning population, where 2 equals 100% of the population before the treatments for each plant. The vertical bars represent the standard error of the mean. * indicates a significant difference compared with the control plants (*P* < 0.05). The values correspond to the mean of 4 plants.

Before starting NAC treatment, the plants already presented chlorotic spots on the leaves, which is characteristic of CVC symptoms. To evaluate the effect of NAC on the bacterial population in infected plants, we used qPCR to estimate the mean of the *X. fastidiosa* cells from at least five replicated plants before and after NAC treatment. Assuming that the initial population (before treatment) was 100%, we estimated the final population after treatment as a percentage of that. In both biological experiments, the number of cells from the NAC-treated plants was significantly lower than from the control 3 months after the beginning of the treatment (Figure 3A). Because qPCR can quantify both living and dead cells, we also performed bacterial isolation in the second experiment to verify the survival of the cells. The number of *X. fastidiosa* cells in plants treated with NAC was lower than the control for all of the evaluated concentrations (Figure 3B), and there was a good correlation between qPCR quantification and bacterial isolation, indicating that part of the population was dead in the plants treated with NAC. Interestingly, in both experiments, the plants not treated with NAC presented more symptomatic leaves than those treated with either 0.48 or 2.4 mg/mL of NAC (Figure S6A–C). A NAC concentration of 6.0 mg/mL was used in the second experiment to test if it would increase the response; however, the plants displayed stunting, their leaves presented a yellow color with an appearance of wilting, and they did not absorb or absorbed very little hydroponic solution during the treatment (Figure S6D), indicating that high concentration of NAC in nutritive solution induced osmotic stress. Because of that, we did not use the NAC concentration of 6.0 mg/mL in further experiments. In addition, in the second biological experiment, we also monitored the plants after interrupting the NAC treatment to verify a possible resurgence of symptoms. Approximately three months after NAC interruption, all of the plants treated with 0.48 and 2.4 mg/mL of NAC started presenting initial CVC symptoms in some leaves (data not shown). These results suggest that to prevent the resurgence of CVC, NAC treatment of the plants needs to be maintained.

#### NAC in solution is absorbed by plant roots

**Figure 4:**
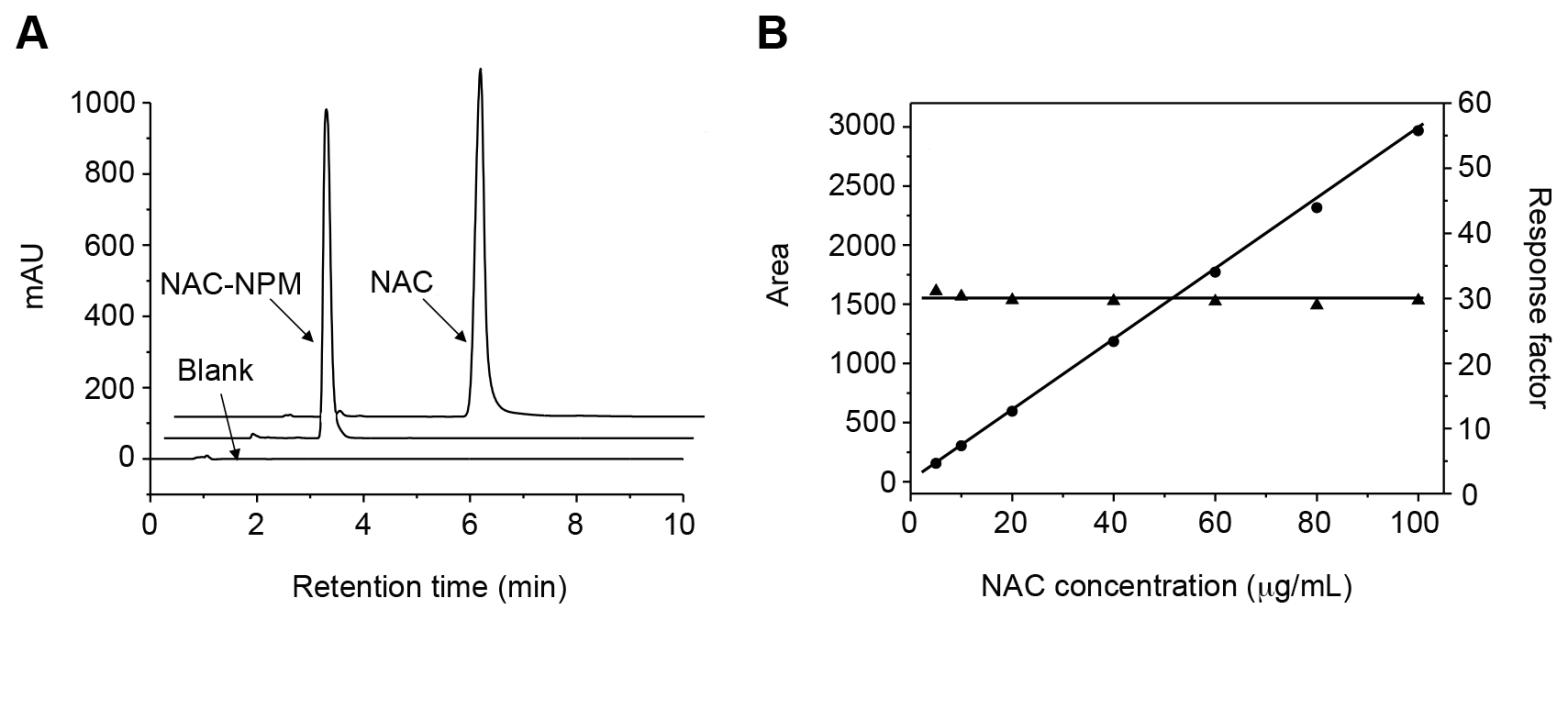
HPLC analysis of NAC. The method specificity among the blank, NAC and NAC-NPM (**A**) and linearity of the quantitative method for NAC-NPM were evaluated by HPLC (**B**); The calibration curve (●) and response factor (▲) were obtained using NAC-NPM standard solutions (*n* = 21). The linearity was assessed through calculating the regression equation (*y* = a*x* ± b) and the correlation coefficient (*r* ^2^) by the least squares method, where: *y* = 29.33*x* + 7.219 and *r* ^2^ = 0.9997. Analytical parameters were as follows: column Phenomenex^®^ Luna C18 (250 x 4,6 mm i.d., 5 μm particle size), Phenomenex^®^ C18 pre-column (4 x 3 mm i.d., 5 μm particle size), mobile phase in a isocratic mode using acetonitrile and formic acid 0.1% (40: 60 v/v), oven temperature at 35°C with a flow rate of 1.2 mL/min, an injection volume of 20 µL and UV detection at 330 nm.

Although NAC is a cysteine analogue that may be easily absorbed by the plants, we used HPLC to certify that indeed the plants absorbed this molecule. We evaluated the NAC concentration in the nutritive solution before and after one week of treatment (time when the solution was fully replaced). To detect NAC using a UV–Vis detector, this compound was derivatized with N-(1-pyrenyl) maleimide (NPM). To check the applicability of the method, parameters such as linearity and range, accuracy and precision, limit of detection and limits of quantitation were investigated (details regarding the results and statistical analyses of these parameters are described in Text S1). The specificity of the method was evaluated by comparing chromatogram analyses of NPM and NAC-NPM samples (Figure 4A). No interfering peak was observed in approximately 3.0 min in the blank chromatogram (retention time of NAC-NPM). The analytical calibration curves (*n = 3*) were linear over the concentration range from 5.00 to 100 µg/mL. The response factor (30.4) showed a slope of -0.0136, which was close to zero and 2.29% of the relative standard deviation among all levels of concentration patterns (Figure 4B). Therefore, the samples could be adequately analyzed after dilution within the concentration range of the proposed method.

The limit of detection and quantitation were calculated in accordance with the standard deviation (sd) (13.81) of the response and slope (*S* = 29.33) of the calibration curve. They were determined to be 1.5 and 4.5 µg/mL, respectively. The results in Table 2 suggest that plants treated with 0.48 and 2.4 mg/mL, but not with 6.0 mg/mL, were able to absorb NAC because the final concentration in these treatments was significantly lower than the initial concentration and the control (NAC in hydroponic solution without plants). The results of using NAC in hydroponic solution were consistent. This method is a promising method for CVC control. Thus, we started new experiments simulating conditions closer to that normally used in the field with plants in the substrate and fertigation as a system to apply NAC.

**Table 2:** NAC quantification via HPLC.

Nominal concentration^a^ (mg/mL)	Measured concentration^b^ (mg/mL)	Standard deviation (St. Dev.)	% of NAC ^c^	% of maximal NAC absorption ^d^
0.48	0.29	0.081	56.9 - 23.1	33.2
2.40	1.98	0.458	36.6 - 0	12.9
6.00	6.16	0.182	0	0

^a^ Initial concentration of NAC that was added to the Leonard jars;

^b^ Concentration of NAC that was measured by HPLC from the samples that were collected from the Leonard jars after one week;

^c^ Absorption values discounting the Standard Dev;

^d^ discounting the NAC amount that was environmentally degraded (measured by NAC solution in Leonard jars without plants).

#### Plants treated with NAC by fertigation displayed CVC remission on leaves with initial symptoms and a reduced bacterial growth rate in plants

**Figure 5:**
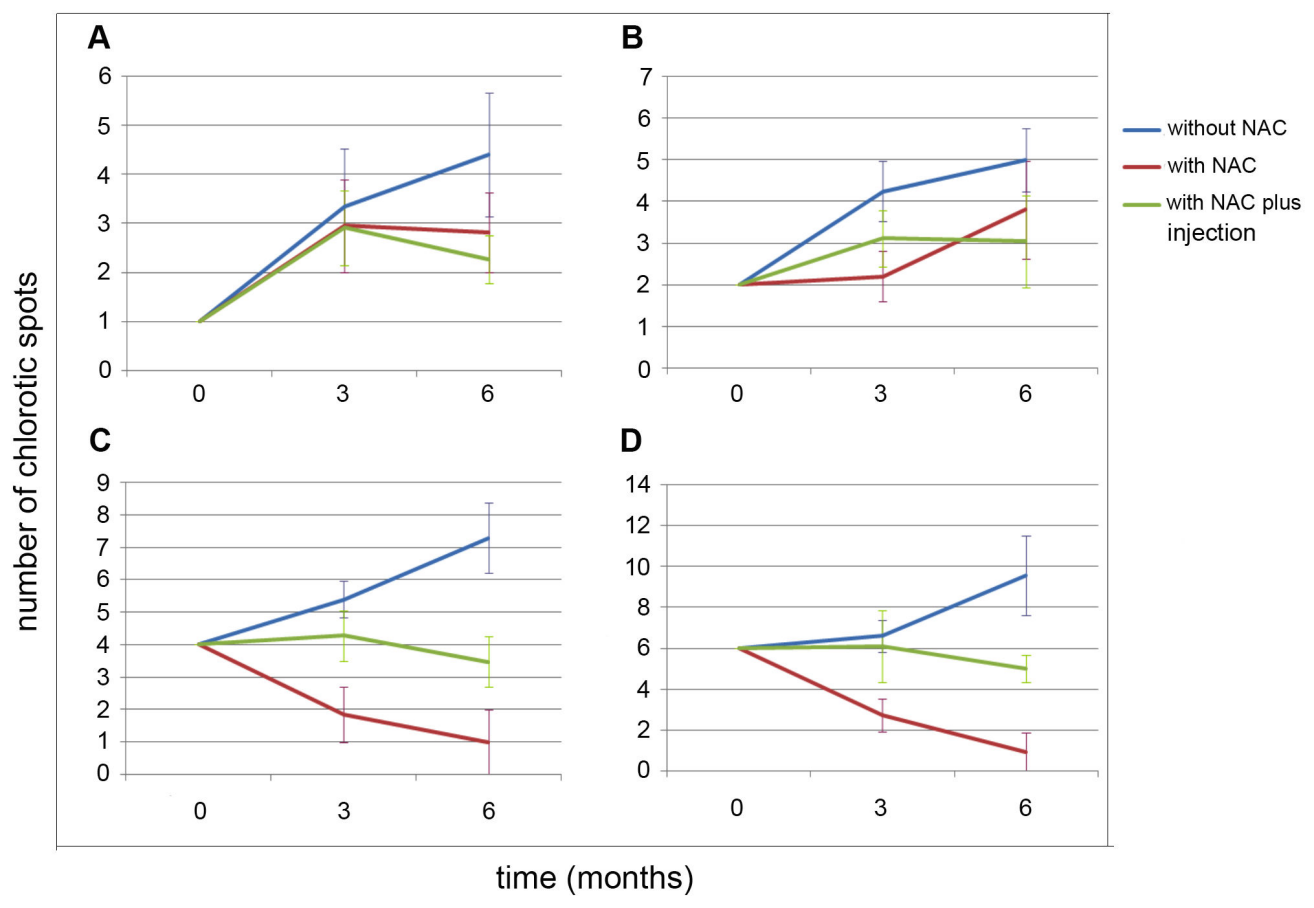
Symptom development in the fertigation experiment. In this experiment, leaves with initial CVC symptoms from untreated plants or treated with NAC by fertigation or fertigation plus injection were compared over time. A, B, C, and D represent the average number of leaves that started the experiment with 1, 2, 4, and 6 chlorotic spots, respectively. The results presented are the averages of at least 10 leaves per plant per treatment. The bars represent the standard deviation of the mean.

**Figure 6:**
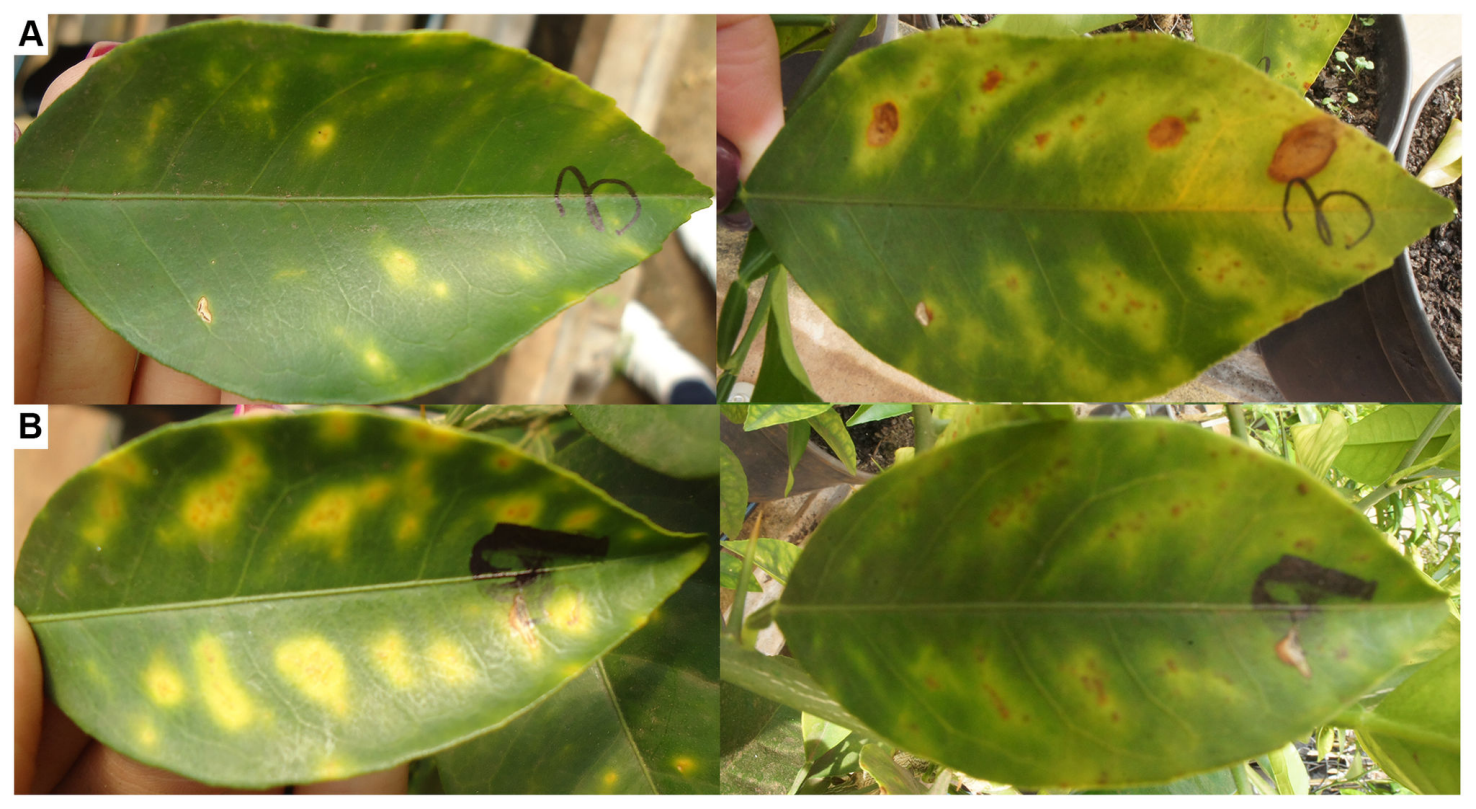
Visual analysis of CVC symptom development. The picture shows representative leaves before and 6 months after starting the NAC treatment. Panel A shows the evolution of the symptoms in an untreated leaf. Panel B shows a leaf with severe symptoms at the beginning and more attenuated symptoms after the treatment.

To test the effect of NAC in CVC control on symptomatic plants, the number of lesions present on individual leaves was monitored for all of the plants at 0, 3, and 6 months after the beginning of NAC treatment. A clear attenuation of symptoms was observed for all the leaves, leading to remission in the cases where the number and severity of lesions was not high (Figure 5). Leaves displaying 1, 2, 4, and 6 chlorotic spots before NAC treatment were monitored at 3 and 6 months after the beginning of the treatment to evaluate the progress of the disease. Overall, for leaves displaying 1 or 2 chlorotic spots, a reduction of symptoms was observed, and no significant difference was observed when NAC was supplemented by injection (Figure 5A-5B). Remission of symptoms was also observed for leaves with 4 and 6 chlorotic spots without any additive effect of NAC added by injection (Figure 5C-5D). These results indicate that the additional application of NAC by injection did not intensify its effect in the plants. When the leaves displayed severe symptoms, such as many chlorotic and necrotic spots, NAC treatment did not reduce the number of counted chlorotic spots but clearly decreased the severity (Figure 6). In general, these results demonstrate that NAC impairs the progression of the disease in plants presenting CVC symptoms, reducing the disease severity and the pace of appearance of new symptomatic leaves. In addition, NAC had no detrimental effect on healthy plants, as they did not present any visual difference compared to the untreated healthy controls (Figure S7).

**Figure 7:**
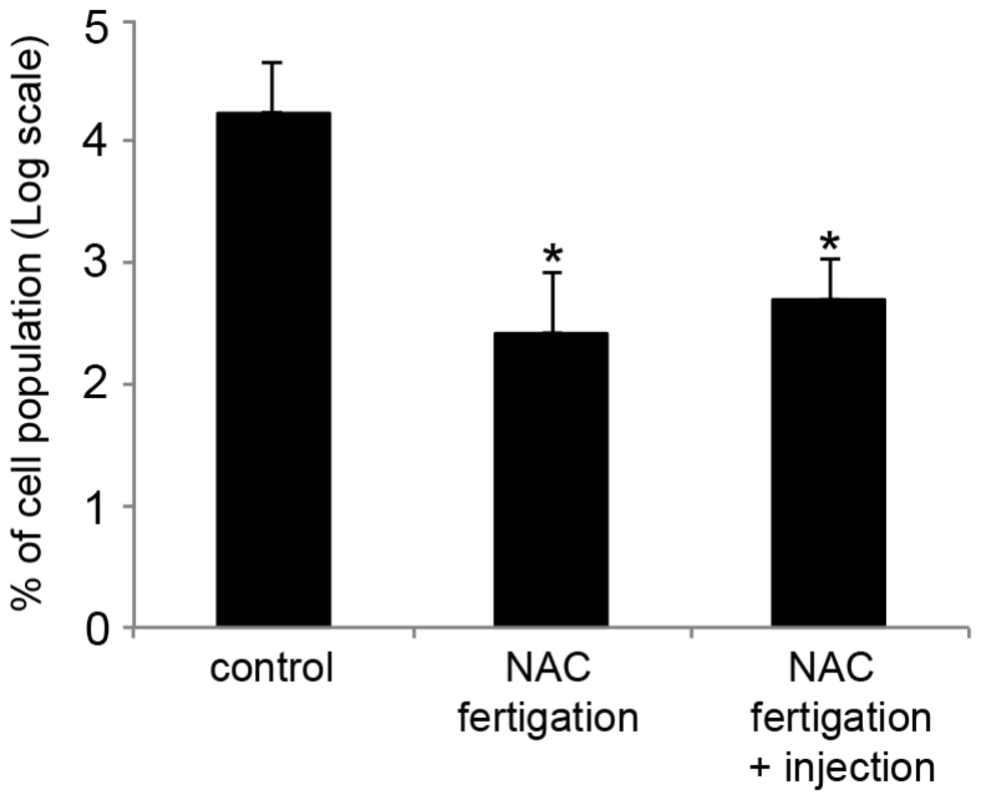
Effect of NAC on *X. fastidiosa* population in fertigation-treated plants. Real-time PCR was used to quantify the bacterial cells in plants treated or not with NAC. The results are represented using a log scale and refer to the percentage of the final population in relation to the beginning population, where 2 equal 100% of the population before the treatments for each plant. The vertical bars represent the standard error of the mean. * indicates a significant difference compared with the control plants (*P* < 0.05). The values correspond to the mean of 5 plants.

The population of the bacteria was evaluated by qPCR at 6 months after the initiation of NAC treatment. As observed in Figure 7, there was an increase in the number of *X. fastidiosa* cells for all of the treatments, but the untreated controls had a higher average number of cells compared with the NAC-treated plants. The total number of *X. fastidiosa* cells in the control increased approximately 100 times, whereas for the plants treated with NAC, the increase was 3 to 5 times. These results suggest that treatment with NAC decreases the growth rate of bacteria in plants. Similar to what was observed in the hydroponics experiment, approximately 3 months after interruption of NAC treatment, the plants started displaying more severe CVC symptoms on the leaves (data not shown). These results reinforce our observation that the plants need to be kept under NAC treatment cycles to prevent the resurgence of CVC symptoms. In addition, we hypothesized that if NAC is applied to the substrate in a system that allows its slow release, and consequently increases its temporal availability to the plant, the intervals of application of NAC in the field could be significantly reduced. To test this hypothesis, we adsorbed NAC in an organic fertilizer known to release slowly in soil.

#### Plants treated with NAC adsorbed on fertilizer reduce CVC symptoms, cell populations and time of symptom resurgence

CVC-diseased plants were pruned to remove all of the symptomatic leaves before starting NAC treatments, after which disease resurgence was followed in the leaves of the new shoots. For the untreated plants, symptoms in the whole plant presented up to 3 months after pruning, whereas for plants treated with NAC-Fertilizer or NAC-Fertilizer plus spray, it took 4 months for development of few symptoms (data not shown). The total number of symptomatic leaves from all the plants was counted at 4 and 6 months after the beginning of the treatment. The number of symptomatic leaves in plants not treated with NAC was significantly higher (*P* < 0.01) at 4 (378 ± 25) and 6 (475 ± 56) months than on plants treated with NAC-Fertilizer (23 ± 11 and 68 ± 35) or NAC-Fertilizer plus spray (21 ± 3.0 and 100 ± 50), at 4 and 6 months, respectively. The amount of symptomatic leaves among the plants treated with NAC-Fertilizer or NAC-Fertilizer plus spray did not significantly differ (*P* > 0.01).

**Figure 8:**
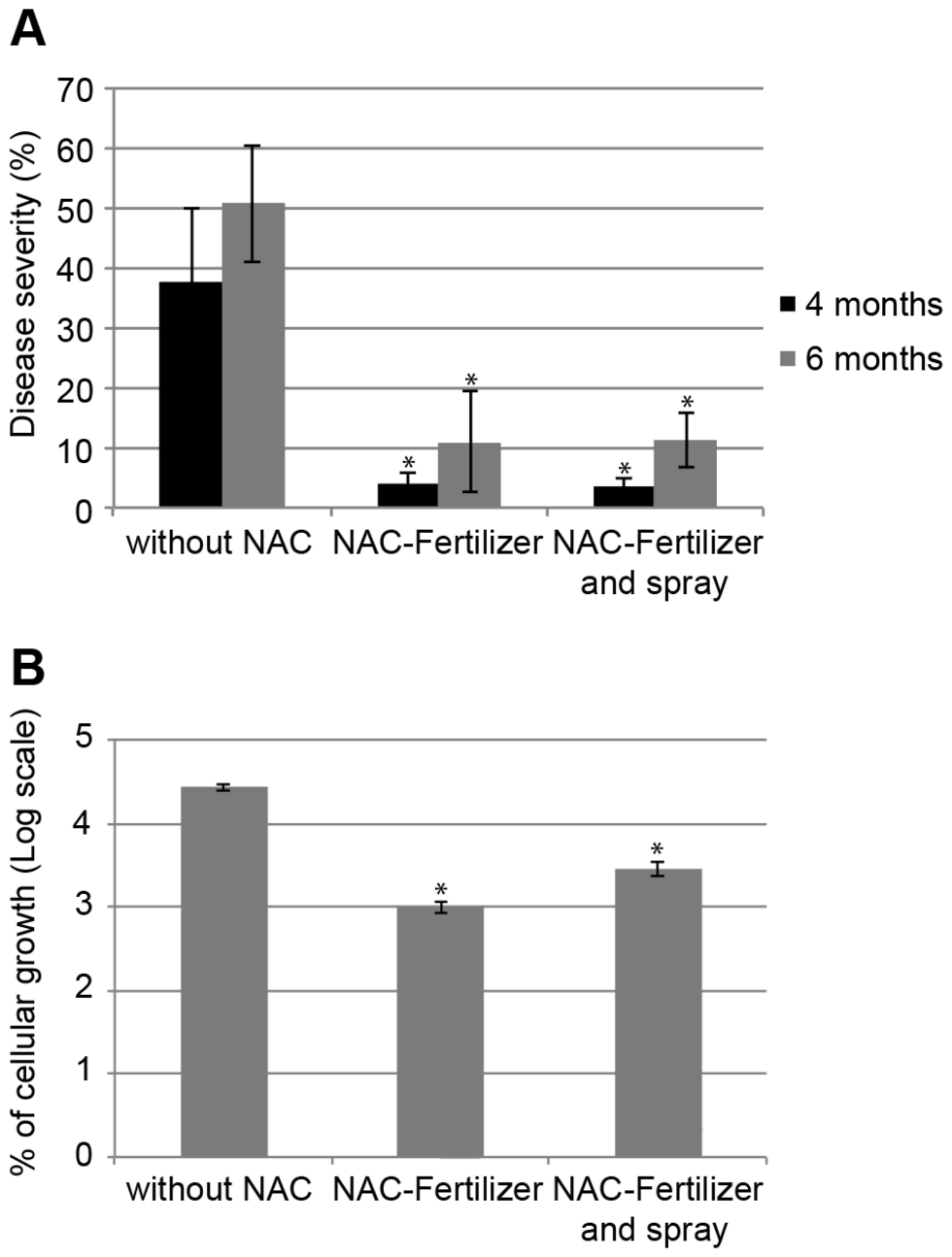
Effect of NAC on severity and bacterial population in plants treated with NAC adsorbed in fertilizer. The severity of disease was evaluated using a diagrammatic scale where 1 and 6 correspond to 3% and 56% of chlorotic areas in a leaf, respectively (A). The *X*. *fastidiosa* population was estimated by real-time PCR analysis (B). The results are represented using a log scale and refer to the percentage of the final population in relation to the beginning, where 2 equals 100% of the population before the treatments for each plant. The vertical bars represent the standard error of the mean. * indicates a significant difference compared with the control (*P* < 0.05). The values correspond to the mean of 5 plants.

**Figure 9:**
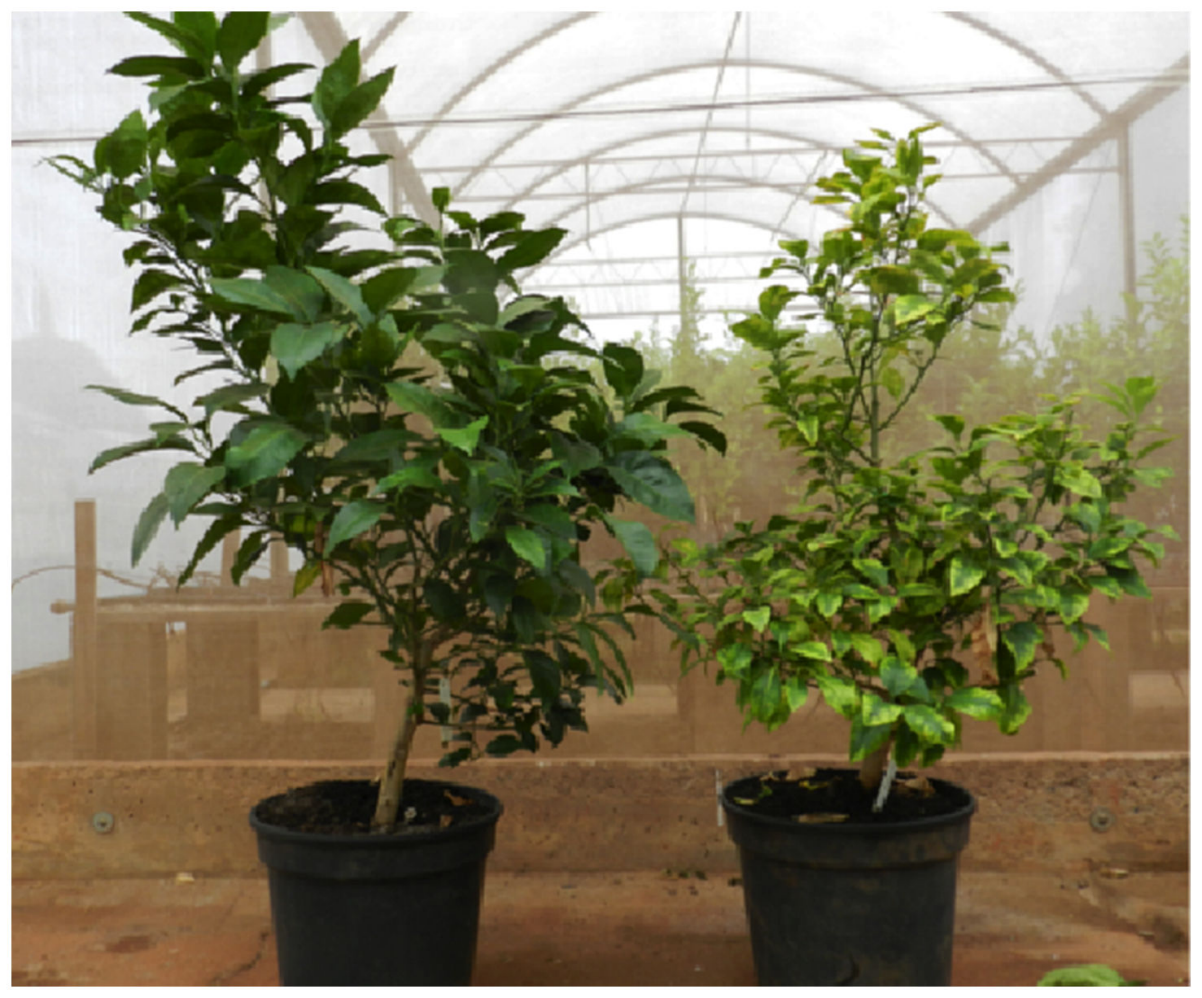
Visual aspect of three-year-old infected plants. The picture shows representative plants that were treated (left) or not (right) with NAC-Fertilizer for 6 months. The picture was taken 8 months after interruption of the NAC treatment.

By using a scale for estimated disease severity (Figure S5), we verified that the untreated plants had an average grade of approximately 5 out of a maximum of 6 (severe symptoms) at 4 months after the beginning of the experiment, evolving to a grade of 6 two months later. On the other hand, for plants treated with NAC-Fertilizer and also NAC-Fertilizer plus spray, the average grade did not reach a score of 2 and 3 on the disease scale, at 4 and 6 months after treatment, respectively (Figure 8A). Therefore, plants treated with NAC displayed less severe symptoms compared with the untreated plants. Furthermore, in untreated plants, the increase in bacterial cell number was more than 250 times, whereas in plants treated with NAC, this increase did not reach a 30-fold increase (Figure 8B), suggesting that treatment with NAC, similar to fertigation treatment, decreases the growth rate of bacteria in plants. In addition, the use of NAC-Fertilizer also delayed the resurgence of symptoms on leaves after interruption of the treatment to approximately 8 months, and the plants presented a better visual aspect than untreated plants (Figure 9). These results indicate that NAC adsorbed in the organic fertilizer is a better way to use this molecule in soil for increasing the intervals of the new application of this product for CVC control.

## Discussion

For a long time, an analogue of cysteine, N-Acetylcysteine (NAC), has been used in medicine as a mucolytic agent to combat many diseases [19–24]. Previous studies have shown that NAC decreases biofilm formation, inhibits bacterial adherence, reduces the production of extracellular polysaccharide matrix, and reduces cell viability for a variety of Gram-negative and Gram-positive bacteria [18,22,25–29]. However, its effect has never been tested on plant–pathogen bacteria. In this study, we tested this molecule against *X. fastidiosa*, a pathogen that affects many different economically important plant species [1]. The strain used in this study causes CVC, a citrus disease responsible for major losses to Brazilian citrus agribusiness.

Our *in vitro* studies demonstrated that concentrations of NAC higher than 1 mg/mL reduced bacterial adhesion to glass surfaces, biofilm formation and EPS production. It has been proposed that NAC could interfere with EPS production in different ways. The sulfhydryl group of NAC is capable of disrupting the disulfide bonds of bacterial enzymes involved in EPS production or excretion through thiol-disulfide exchange [29]. Moreover, because of the antioxidant property of NAC, it can indirectly affect bacterial cell metabolism and EPS production [18,22]. In addition to the effect on EPS, NAC could also impair the proper function of the bacterial adhesion proteins and consequently decrease the biofilm [27,29]. Therefore, this effect could explain the results observed in the *in vitro* experiments. We also verified a decrease of *X. fastidiosa* viability upon NAC treatment. Growth reduction with 1 mg/mL of NAC was higher than 80%, and no growth was observed with 6 mg/mL. This concentration is much lower than what is necessary to inhibit human bacterial pathogens, i.e., for *Haemophilus influenzae* and *Streptococcus pneumoniae*, the concentrations are 10 and 50 mg/mL, respectively [40], for *Pseudomonas aeruginosa* from 10 to 40 mg/mL [27], for *Helicobacter pylori* 15 mg/mL [26], for *Enterococcus faecalis* 12.5 mg/mL [29], and 80 mg/mL for a wide range of other clinical pathogens [28].

The pathogenicity of *X. fastidiosa* involves biofilm formation and movement through the xylem vessels of the host plant. Both characteristics require specific bacterial adhesins that have an important role in aggregation and motility [8,34]. It has been suggested that thiol groups could be active on the surface of *X. fastidiosa* proteins and appear to promote cell-cell aggregation by forming disulfide bonds with thiol groups on the surface of adjacent cells [41]. Because many adhesion proteins of *X. fastidiosa* contain cysteine residues that can form disulfide bounds and promote aggregation, we speculate that NAC could avoid the formation of disulfide bounds, preventing the attachment of the cells and therefore the biofilm formation. In addition, because of the chemical nature of NAC with its reactive–SH group, irreversible damage could be done to a large number of bacterial proteins that are essential for growth and metabolism [42].

As *in vitro* experiments showed promising results, studies *in planta* were performed with the aim of using NAC for controlling *X. fastidiosa*. Thereafter, our work focused on conditions where the plant was already infected with the bacterium to verify if this molecule has a bactericidal effect in the plant as observed in the *in vitro* experiments. NAC was applied to the root system with the expectation that it could promptly reach the target bacterium because *X. fastidiosa* lives in xylem, and any compound absorbed by the root goes straight to these vessels. The clear differences in the phenotype of plants treated or not with NAC support the idea that it was translocated and had an effect inside the plants. In addition, HPLC analysis indicated that NAC at 0.48 mg/mL and 2.4 mg/mL is absorbed by the plants because the amount of NAC in solutions with plants was lower than in control solutions after one week. In addition, these concentrations were able to decrease the symptoms and bacterial population in plants in the hydroponic system. On the other hand, a NAC concentration of 6.0 mg/mL induced osmotic stress with plants showing typical phenotypes like stunting, wilting, and incapacity of absorbing the nutritive solution. These results indicate that there is an effective range of NAC concentration useful for treating plants.

Other experiments were performed in plants to simulate conditions closer to the field, and therefore, substrate was used for plant growth. First, NAC was applied by fertigation, and fertigation plus injection in xylem tissue was conducted to maximize its effect in xylem vessels. However, for both experimental conditions, we obtained similar results. NAC was able to decrease the initial symptoms on the leaves and to retard bacterial growth in plants. On the other hand, the bacterial population levels observed for fertigation were not the same as observed for the hydroponic experiments. For the fertigation experiments, the bacterial population increased even in NAC treatment but much less than in the controls, whereas in the hydroponic system, the bacterial population decreased after NAC treatment. The plants used in the fertigation experiments were older than 2 years of age and presented a much more established population of bacteria. These factors could explain this difference in the growth of bacterial cells in both experiments.

Three months after interrupting the NAC treatment, symptom resurgence was observed in both plants grown in hydroponic systems and those treated by fertigation. These results, together with those showing that the bacteria were not dead in the plant, suggest that NAC has some inhibitory effect on the bacteria and affects the disease symptoms, but this effect is reversible and depends on the presence of NAC in the plants. Thus, we proposed that the method used to provide NAC to the plants could be improved because HPLC experiments demonstrated environmental degradation of NAC in solution. In addition, when NAC is applied by fertigation, part of this molecule can be retained in the soil and possibly a small fraction of NAC is indeed available to the plants. To improve the NAC availability to the plant, this molecule was adsorbed in an organic fertilizer known to display slow nutrient release in field conditions. In these experiments, in addition to NAC application in the substrate, we also sprayed NAC on the leaves. This second method was performed to verify if NAC could have beneficial effects for the plant other than inducing damage to the bacteria. If so, this treatment would display better results than NAC application merely in the root. However, we did not observe any significant difference among the treatments, suggesting that the application of NAC to the root system is the best and most economical method of achieving *X. fastidiosa* control. Nevertheless, spraying NAC to control phytopathogenic bacteria that colonize phloem vessels or the mesophyll tissue, for instance, could be a good control strategy but remains to be further tested.

The use of NAC-Fertilizer led to greatly reduced symptoms and lower bacterial populations in plants compared with controls without NAC. In addition, a longer delay in symptoms resurgence after NAC interruption (approximately 8 months) was observed compared with the other NAC treatments (approximately 3 months) used in this work. These results demonstrate that NAC-Fertilizer most likely increased the time of NAC availability to the plant and consequently decreased the damage of CVC disease. Thus, NAC-Fertilizer or any other compound that would allow a slow release of NAC might be a useful real-world strategy that could be applied in the field for controlling CVC. In this work, we used plants that were already infected with *X. fastidiosa* to verify the effect of NAC on this bacterium; however, NAC treatment could also be a promising strategy for the prevention of bacterial infection, but this hypothesis needs to be further investigated.

Because NAC can reverse the symptoms in diseased plants as shown in our results, we speculate that application of NAC could also be very beneficial in some field situations. First, it is known that plants host this bacterium without developing symptoms and that stress conditions could trigger the disease [43]. Second, the development of the initial CVC symptoms is not enough to cause fruit damage. In these cases, we suggest that the use of NAC could retard the progression of the disease as long as the NAC treatment is maintained in the plants in contrast to, for instance, pruning where symptom development is just delayed until a later time when the plant is not productive anymore. In addition, NAC could be applied to plants just in the periods when there is an increase of bacterial transmission by the insect vector. We do not know the effect of insect transmission on plants treated with NAC; however, as NAC most likely affects biofilm formation, we speculate that transmission would also be compromised in NAC-treated plants, since the insect vector acquires *X. fastidiosa* cells preferentially when they are in biofilm [6], but this hypothesis needs to be further tested.

There is a good potential for the use of NAC as an antimicrobial molecule in phytopathogen control. NAC is a small molecule that experiences environmental degradation in solution, making it more sustainable for use in the environment than other products commonly used in agriculture. In addition, NAC has already been used in humans for medical purposes for a long period, and studies concerning its effects in humans indicate that it is not damaging to human health [20]. NAC adsorbed in organic fertilizer is an economically promising alternative compared with the current management of CVC, which includes pruning, use of insecticide and eradiation of severe symptomatic plants [2,9]. Previously published studies also indicate a synergistic effect for NAC when used together with other substances [27]. Therefore, the use of NAC can be improved to potentiate its effect for agricultural proposes. This work is a pioneer in the study of NAC against phytopathogenic bacteria, and it opens real possibilities for future studies aimed at the control of other *X. fastidiosa* strains as well as bacteria associated with diseases in different crops.

## Supporting Information

Figure S1 **Sweet orange plants infected with *X. fastidiosa* by grafting.**
Branch with CVC symptoms (A). Tongue approach grafting that allows the scion donor-plant to remain on the rootstock until the graft heals (B and C). Overview of seedlings grafted on a *X. fastidiosa* donor plant (D).
(TIF)

Figure S2 **Leonard jar preparation (A) and hydroponic experiment before NAC treatment showing all of the plants with CVC symptoms (B).**
(TIF)

Figure S3 **General view of the fertigation experiment.**
Fertigation tank (A) and system of NAC solution application (B). Steps of NAC application by syringe (C). The stems of the plants were drilled (1), a 200 µL tip fixed with a rubber was put into the hole (2), and a syringe containing 10 mL of the NAC solution was connected to the rubber (3), allowing the injection of the solution.
(TIF)

Figure S4 **General view of the NAC-Fertilizer experiments.**
Fertilizer without (A and C) and with NAC (B and D) was applied on the substrate (A and B) and homogenized with it (C and D). NAC application by spray (E).
(TIF)

Figure S5 **Diagrammatic scale for evaluation of severity of Citrus Variegated Chlorosis (CVC) on adaxial (A) and abaxial (B) leaf surfaces.**
Scores of 1, 2, 3, 4, 5, and 6 correspond to 3, 6, 15, 25, 35, and 56% of leaf area with chlorosis, respectively, based on Amorim et al., 1993.
(TIF)

Figure S6 **Effect of NAC in hydroponics.**
General view of representative plants from untreated control showing CVC symptoms (**A**), NAC treatment of 0.48 mg/mL (**B**), 2.4 mg/mL (**C**), and 6.0 mg/mL (**D**). The images were obtained 3 months after the beginning of NAC treatment. The arrows indicate the CVC symptoms.
(TIF)

Figure S7 **Representative plants showing that NAC had no detrimental effect on healthy plants, either when NAC was added by fertigation (A) or when in addition to fertigation, NAC was injected (B), compared with the untreated healthy control (C).**
(TIF)

Text S1 **NAC quantification by HPLC.**
(DOCX)
