# Supplementary figures and images for: N-Acetylcysteine in Agriculture, a Novel Use for an Old Molecule: Focus on Controlling the Plant–Pathogen Xylella fastidiosa
Source: PLoS One. 2013 Aug 23;8(8):e72937. doi: 10.1371/journal.pone.0072937 (PMC3751844; doi:10.1371/journal.pone.0072937)

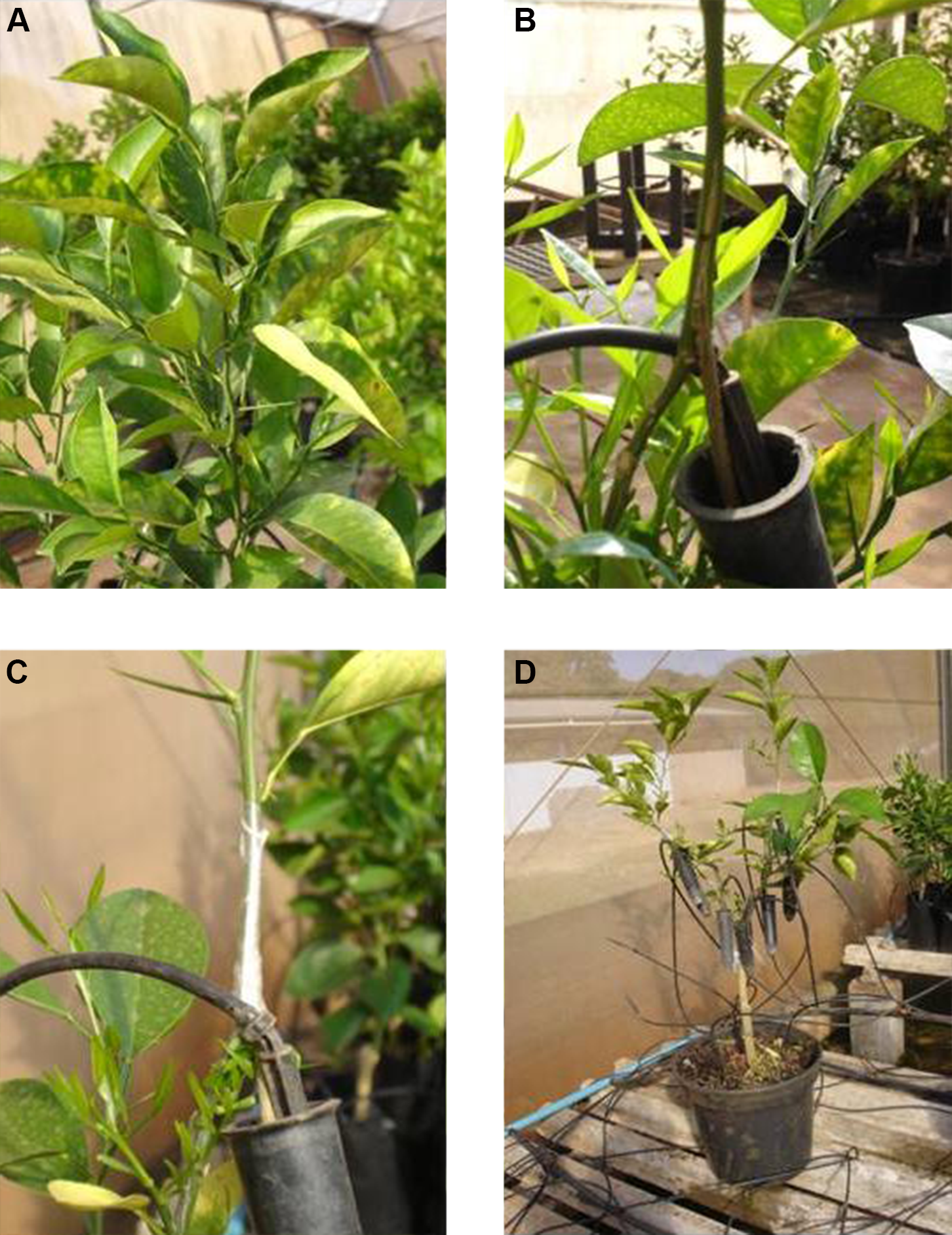

Supplement: Figure S1 — Sweet orange plants infected with X. fastidiosa by grafting. Branch with CVC symptoms (A). Tongue approach grafting that allows the scion donor-plant to remain on the rootstock until the graft heals (B and C). Overview of seedlings grafted on a X. fastidiosa donor plant (D). (TIF) [file pone.0072937.s001.tif]

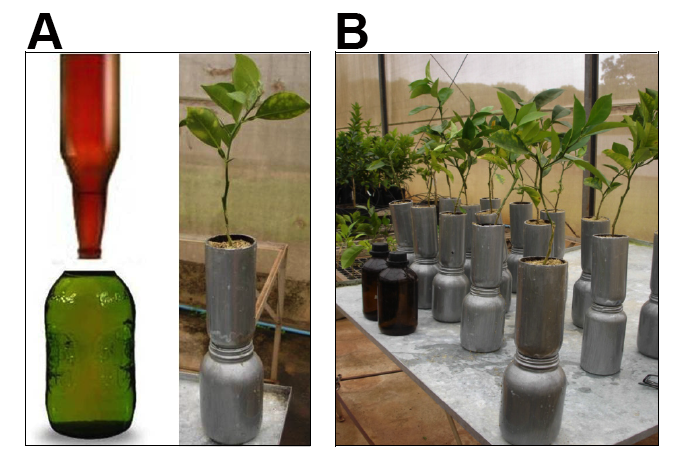

Supplement: Figure S2 — Leonard jar preparation (A) and hydroponic experiment before NAC treatment showing all of the plants with CVC symptoms (B). (TIF) [file pone.0072937.s002.tif]

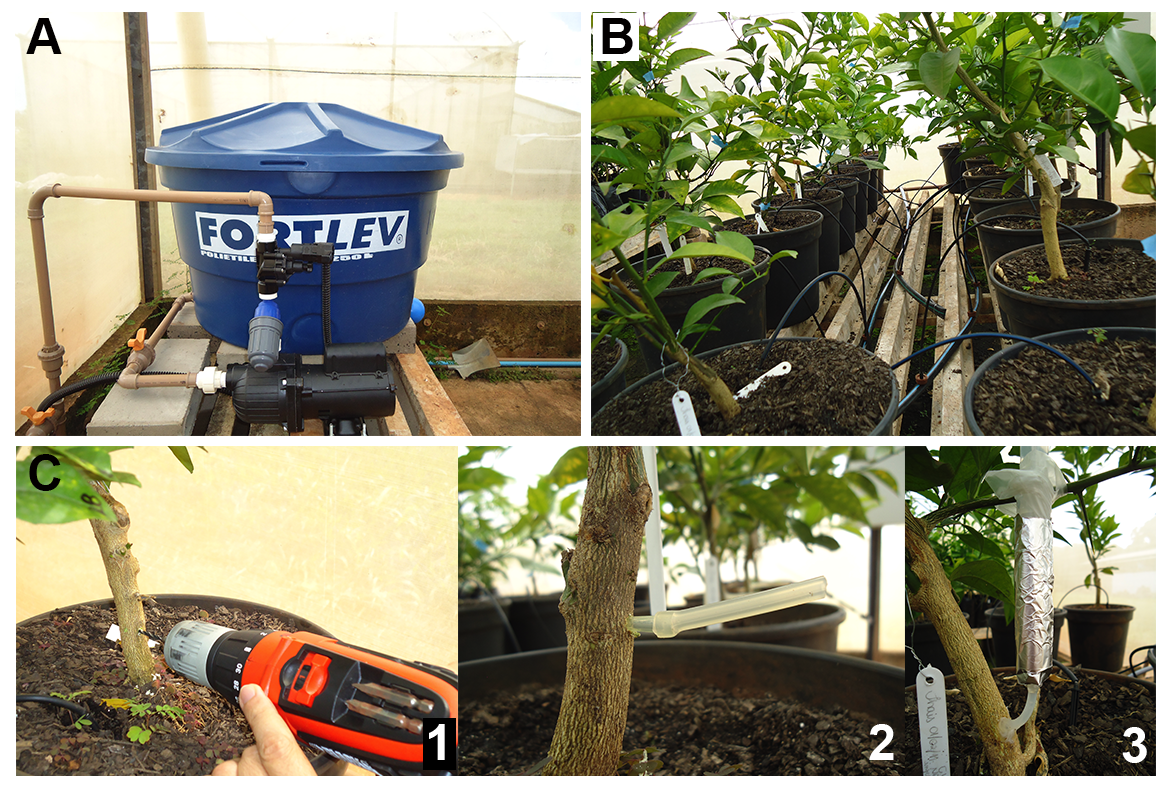

Supplement: Figure S3 — General view of the fertigation experiment. Fertigation tank (A) and system of NAC solution application (B). Steps of NAC application by syringe (C). The stems of the plants were drilled (1), a 200 µL tip fixed with a rubber was put into the hole (2), and a syringe containing 10 mL of the NAC solution was connected to the rubber (3), allowing the injection of the solution. (TIF) [file pone.0072937.s003.tif]

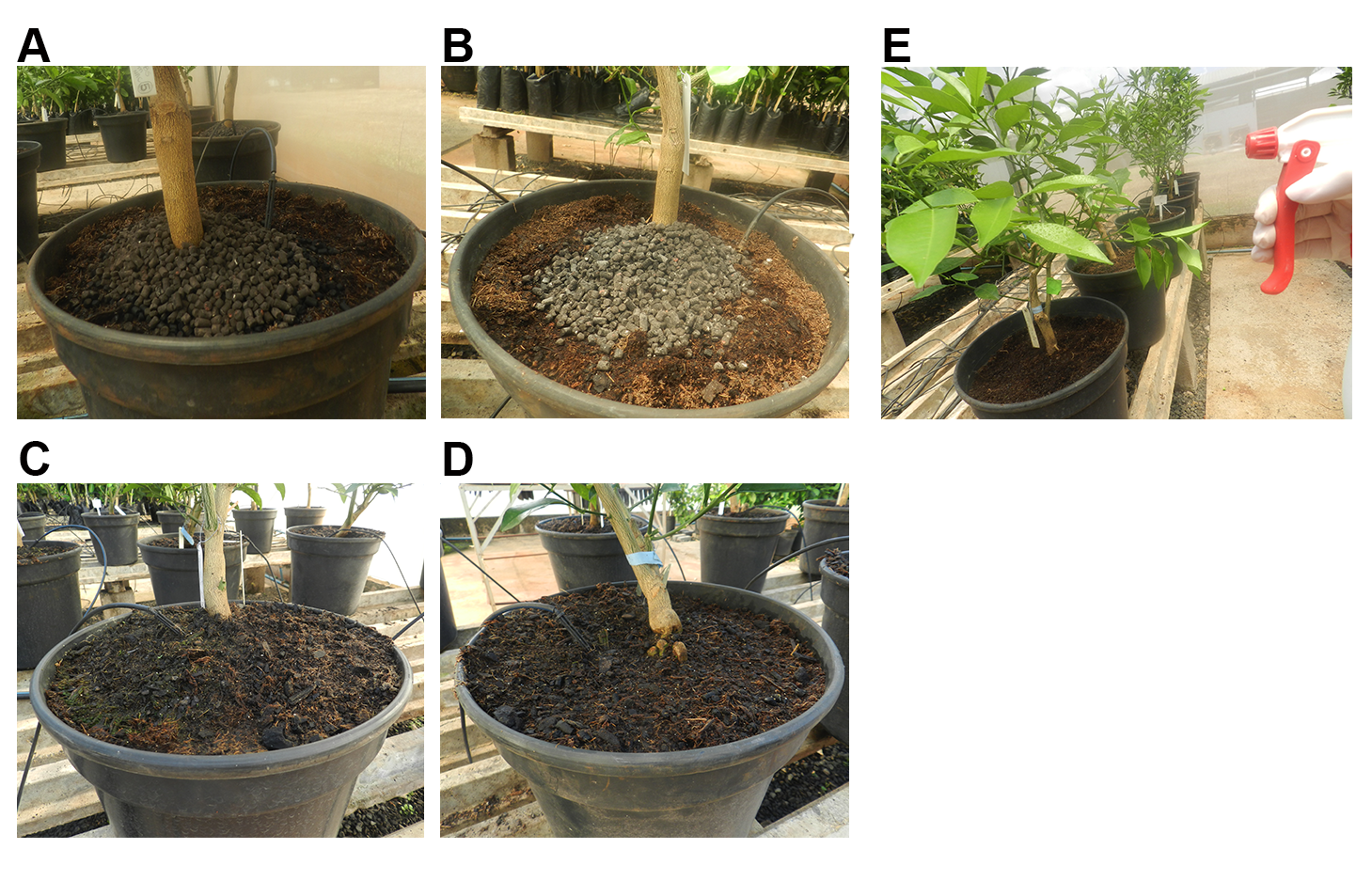

Supplement: Figure S4 — General view of the NAC-Fertilizer experiments. Fertilizer without (A and C) and with NAC (B and D) was applied on the substrate (A and B) and homogenized with it (C and D). NAC application by spray (E). (TIF) [file pone.0072937.s004.tif]

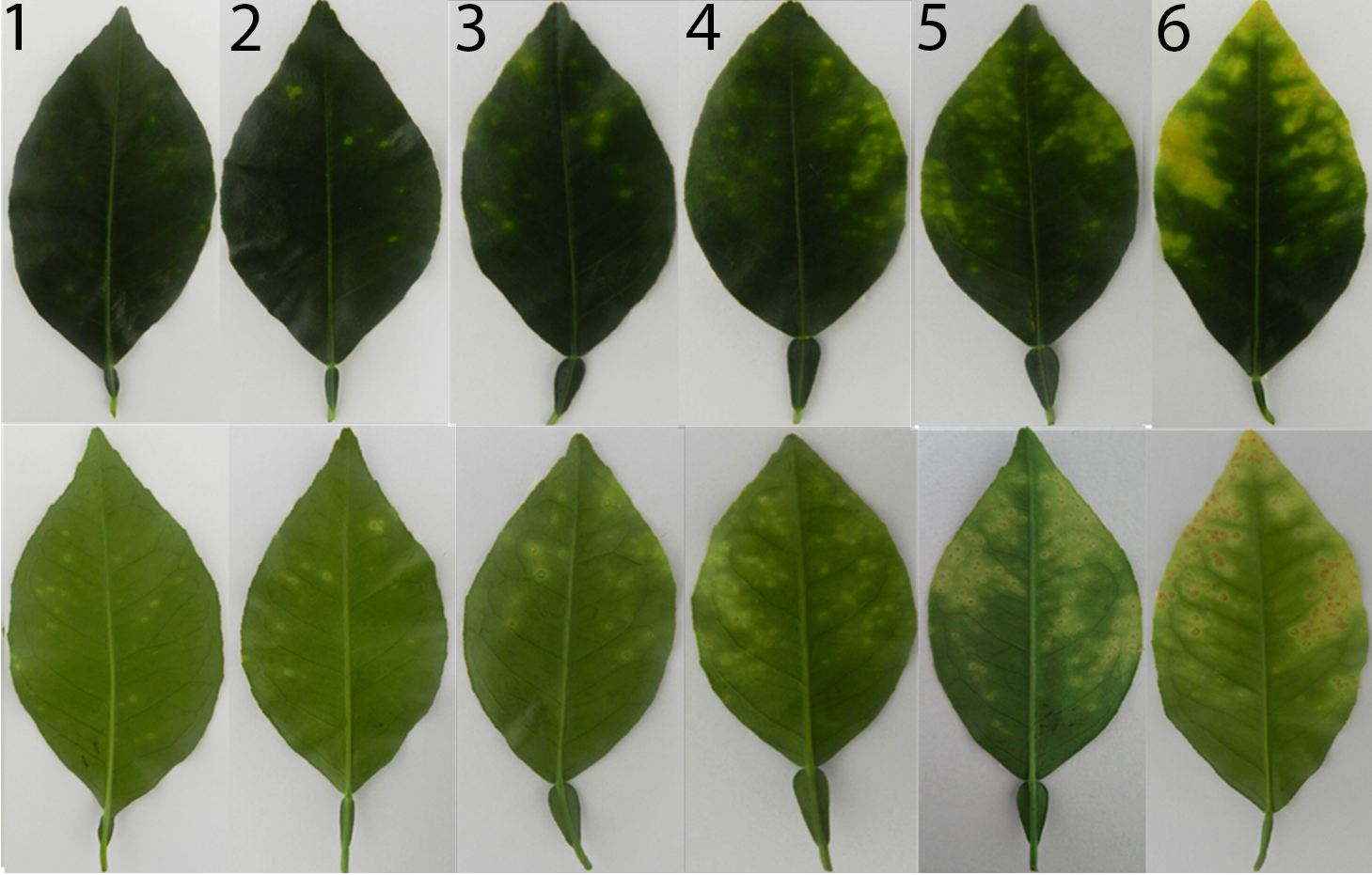

Supplement: Figure S5 — Diagrammatic scale for evaluation of severity of Citrus Variegated Chlorosis (CVC) on adaxial (A) and abaxial (B) leaf surfaces. Scores of 1, 2, 3, 4, 5, and 6 correspond to 3, 6, 15, 25, 35, and 56% of leaf area with chlorosis, respectively, based on Amorim et al., 1993. (TIF) [file pone.0072937.s005.tif]

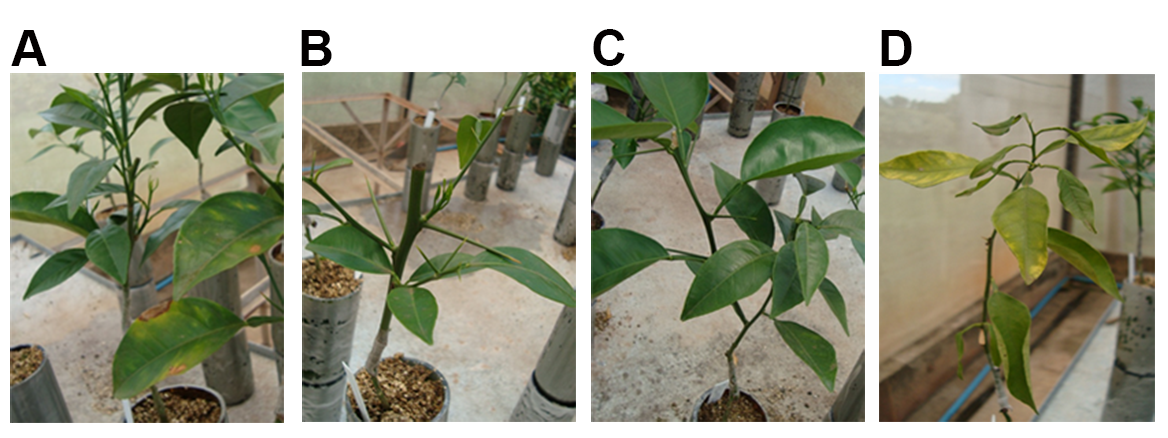

Supplement: Figure S6 — Effect of NAC in hydroponics. General view of representative plants from untreated control showing CVC symptoms (A), NAC treatment of 0.48 mg/mL (B), 2.4 mg/mL (C), and 6.0 mg/mL (D). The images were obtained 3 months after the beginning of NAC treatment. The arrows indicate the CVC symptoms. (TIF) [file pone.0072937.s006.tif]

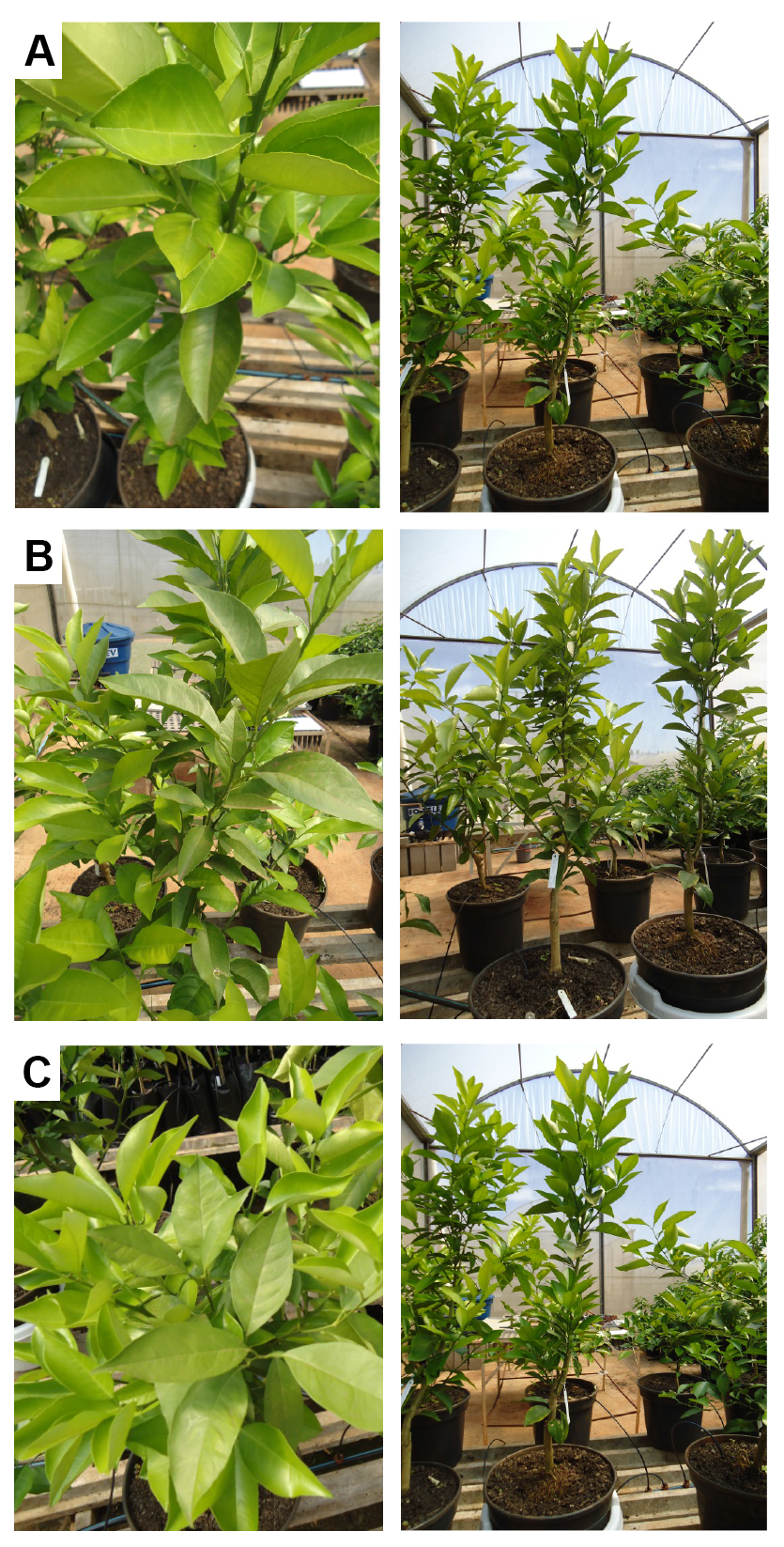

Supplement: Figure S7 — Representative plants showing that NAC had no detrimental effect on healthy plants, either when NAC was added by fertigation (A) or when in addition to fertigation, NAC was injected (B), compared with the untreated healthy control (C). (TIF) [file pone.0072937.s007.tif]
