# Supplementary material for: N-Acetylcysteine in Agriculture, a Novel Use for an Old Molecule: Focus on Controlling the Plant–Pathogen Xylella fastidiosa
Source: PLoS One. 2013 Aug 23;8(8):e72937. doi: 10.1371/journal.pone.0072937 (PMC3751844; doi:10.1371/journal.pone.0072937)
Supplement: Text S1 — NAC quantification by HPLC. (DOCX) [file pone.0072937.s008.docx]

**NAC quantification by HPLC**

**Preparation of the calibration solutions and samples derivatizations**

A stock solution was prepared by accurately dissolving 1.6 mg of NAC into 10 mL of nutritive solution. Seven standard solutions (5.00, 10.0, 20.0, 50.0, 60.0, 80.0, and 100 µg/mL), each one in triplicate, were obtained by diluting the stock solution in sufficient amount of nutritive solution to a final volume of 1 mL. These solutions were used to perform the analyses of calibration curve, linearity, range, limit of detection (LOD) and limit of quantification (LOQ) of the method.

In order to detect NAC by using of UV-Vis detector, this compound was derivatized with N-(1-pyrenyl) maleimide (NPM) according to Wu et al. [1]. Therefore, 100 µL of each sample were added to 300 µL of the NPM solution and incubated at 60^o^C for 1 hour; then 10 µL of 2M chloridric acid (HCl) was added to stop the derivatization reaction. The final pH of the solutions was below 2.0, which is important for stabilizing the derivative NAC compound (**NAC-NPM**). Derivatized samples were injected directly into the HPLC system. All standards and samples were treated with NPM before HPLC analyses.

**Calibration curve and method validation**

The validation of the analytical method was carried out according to the criteria proposed by the International Conference on Harmonization of Technical Requirements for Registration of Pharmaceuticals for Human Use [2]. Linearity and range were evaluated by constructing a calibration curve using peak areas versus nominal concentrations of **NAC-NPM** from 5 to 100 µg/mL. Linear regression analysis was carried out by plotting peak area (*y*) versus **NAC-NPM** concentration (*x*), and through the analysis of respective response factors (i.e. the peak area divided by the concentration of each standard sample) and residuals. In order to confirm the analytical method validation, a regression analysis of variance (ANOVA) of the linear regression data measurements was performed evaluating the significance of the proposed method. Statistical significance was established at the *P*-value <0.05, which indicates that the model is explained by the proposed regression at a 95% confidence interval.

Specificity was evaluated by comparing the chromatograms from samples containing possible interfering substances (e.g., samples of nutritive solutions, free of NAC and/or NPM, blanks) and samples of derivative compounds. Tests to determine the accuracy and precision were performed using solutions of low, medium and high concentrations (6.00, 42.0, and 90.0 µg/mL) of **NAC-NPM** standards, each one covering the entire linearity range. Accuracy was determined by calculating the recovery percentage of the average from NAC solutions (*n*=5). It was determined the ratio of standard deviation (*sd*) in three nonconsecutive days (*n=3*). Precision of the assay was determined by repeatability (intra-day) and intermediate precision (inter-day) and reported as Relative Standard Deviation (RSD) for a statistically significant number of replicate measurements. A total of 15 samples for each evaluated concentration level were prepared, being five analyzed per day. The limits of quantification (LOQ) and detection (LOD) were calculated mathematically by the relationship between the standard deviation (*sd*) of the calibration curve and its slope (*S*) using the multiplier suggested by the ICH standard (International Conference on Harmonization of Technical Requirements for Registration of Pharmaceuticals for Human Use). The LOD and LOQ were calculated from the following equations: LOD = (3.3 x *sd / S*) and LOD = (10 x *sd / S*).

The repeatability of the method was investigated by evaluating the correlation between the results of successive measurements of the same sample of derivative NAC that was analyzed under the same conditions for monitoring the analyst, equipment and place in a short time. The autosample stability was measured by determining the concentration of standard samples kept in HPLC autosampler vial and stored at room temperature every 2h for 24h.

**Results**

**Calibration curve and method validation**

The analytical calibration curves (*n = 3*) were linear over the concentration range from 5.00 to 100 µg/mL (**Figure 10a**). The linearity was assessed through calculating the regression equation (*y* = ax ± b) and the correlation coefficient (*r^2^*) by the least squares method, where: *y* = 29.33*x* + 7.219 and *r^2^* = 0.9997. The *y* is the area of chromatographic peak, and *x* the concentration of standard solution in µg/mL. The standard deviations of the values of *a* and *b* are indicated in **Table 1**. Correlation coefficient showed a *P*-value < 0.05 by variance analysis (**Table 1**). When *r^2^* values are greater than 0.999 it indicates that there is a good correlation of linearity through all the concentrations used and a homoscedastic distribution of replicates at all levels that were applied in the calibration curve assembly. The ANOVA output of the linear regression model described in **Table 1** also confirms the linearity and sensibility of the model. It was observed that the slope of calibration curve was very significantly different from zero (*P*-value < 0.05) in accordance with a high sensibility for the method.

**Table 1.** Summary of the output of the ANOVA for the linear regression analysis.

| **ANOVA** |  |  |  |  |  |  |
| --- | --- | --- | --- | --- | --- | --- |
|  | ***d.f.*** | ***SS*** | ***MS*** | ***F*** | ***P-value*** |  |
| Regression | 1 | 2.05x10^7^ | 2.05x10^7^ | 5.95x10^4^ | 1.12x10^-34^ |  |
| Residual | 19 | 6.56x10^3^ | 345.0 |  |  |  |
| Total | 20 | 2.05x10^7^ |  |  |  |  |
|  |  |  |  |  |  |  |
|  | ***Coefficients*** | ***S.E*** | ***t-Stat.*** | ***P-value*** | ***Lower 95%*** | ***Upper 95%*** |
| Intercept (*a*) | 7.219 | 6.762 | 1.067 | 0.299 | -6.934 | 21.37 |
| Slope (*b*) | 29.33 | 0.120 | 243.8 | 1.12x10^-34^ | 29.08 | 29.58 |

The accuracy was analyzed by calculating the average percentage of recoveries for **NAC-NPM** at three different concentrations. The three standard solutions (6.00, 50.0 and 90.0 µg ml^-1^) were each one carefully prepared in quintuplicate and analyzed by the proposed method in three nonconsecutive days (*n = 3*). The same solutions were used to calculate the precision. The total average recovery (accuracy) and its RSD found was 99.3 ± 2.95%, showing strong agreement between the experimental and theoretical values. The precision was represented by Relative Standard Deviation (RSD). RSD for repeatability at each concentration level of standard solutions intra-day (*n = 15*) and inter-day (*n = 3*) were lower than 1.17% and 1.76%, respectively. The results indicate good precision of the analytical method. Detailed results for the three concentration levels which were tested are shown in **Table 2**.

**Table 2.** Results of precision for three different concentrations of **NAC-NPM**.

| **Nominal concentrations (µg ml^-1^)** | **Mean^a^** | **Day** | **RSD (%)** |
| --- | --- | --- | --- |
| Intra – Day Variation (*n* = 5 each level) | | | |
| 6.00 | 5.69 | 1 | 0.001 |
| 50.0 | 50.8 | 1 | 1.17 |
| 90.0 | 91.7 | 1 | 1.02 |
| 6.00 | 5.87 | 2 | 1.06 |
| 50.0 | 49.2 | 2 | 0.512 |
| 90.0 | 92.2 | 2 | 0.802 |
| 6.00 | 5.72 | 3 | 1.41 |
| 50.0 | 49.5 | 3 | 1.35 |
| 90.0 | 91.9 | 3 | 0.490 |
| Inter – Day Variation (*n* = 15 each level, 3 days) | | | |
| 6.00 | 5.76 | - | 1.45 |
| 50.0 | 49.8 | - | 1.76 |
| 90.0 | 91.9 | - | 0.83 |

*^a^ Mean found concentration (µg ml^-1^).*

**References**

1. Wu W, Goldstein G,Adams C, Mafthewsa RH, Erca N (2006) Separation and quantification of N-acetyl-L-cysteine and N-acetyl-cysteine-amide by HPLC with fluorescence detection. Biomedical Chromatography 20: 415-422.
2. International Conference on Harmonization, FDA, USA. Q2B: Validation of analytical procedures: methodology. 1996. URL

(http://www.fda.gov/downloads/Drugs/GuidanceComplianceRegulatoryInformation/Guidances/UCM073384.pdf)(accessed march 10th 2013).
